# Comparison of Two Commercially Available Immunoassays for the Measurement of Bovine Cardiac Troponin I in Cattle With Induced Myocardial Injury

**DOI:** 10.3389/fvets.2020.00531

**Published:** 2020-08-27

**Authors:** Joe S. Smith, Anita Varga, Karsten E. Schober

**Affiliations:** ^1^Department of Veterinary Diagnostic and Production Animal Medicine, College of Veterinary Medicine, Iowa State University, Ames, IA, United States; ^2^Department of Biomedical Sciences, College of Veterinary Medicine, Iowa State University, Ames, IA, United States; ^3^Gold Coast Veterinary Service and Consulting, Esparto, CA, United States; ^4^Department of Veterinary Clinical Sciences, College of Veterinary Medicine, The Ohio State University, Columbus, OH, United States

**Keywords:** cardiac troponin I, cattle, ionophore, i-STAT, monensin, toxicity

## Abstract

**Background:** Multiple cardiac troponin I (cTnI) immunoassays are commercially available. Overall, assays have not been standardized, and inter-assay differences in the detection of the analyte cardiac troponin I can be clinically relevant.

**Objective:** To compare the diagnostic accuracy of the commercially available Abbott i-STAT®1 cTnI immunoassay (i-STAT) and the previously validated ADVIA Centaur TnI-Ultra immunoassay (Centaur) in cattle.

**Hypothesis:** There will be significant differences in bovine serum cTnI results measured by the Centaur and i-STAT methods.

**Animals:** Ten dairy cows with experimentally induced myocardial injury due to monensin administration. Thirty apparently healthy dairy cows with no history of monensin exposure served as controls.

**Methods:** Blood was collected at various time points after administration of a single dose of monensin (20 to 50 mg/kg) via orogastric tube. A total of 112 blood samples were collected. Cardiac TnI concentration was analyzed with the two methods and the association between methods analyzed via linear regression. Bland-Altman analysis to evaluate agreement between methods was performed on samples divided into groups (cTnI < 1.0 ng/mL and cTnI ≥ 1.0 ng/mL).

**Results:** Analyzer results were linearly correlated with each other (*R*^2^ = 0.931). Samples with cTnI concentrations <1.0 ng/mL had a bias of −0.13 ± 0.20 ng/mL and samples with cTnI concentrations >1.0 ng/mL had a bias of −9.81 ± 13.26 ng/mL.

**Conclusions and clinical importance:** The results of this study reveal that cTnI concentrations determined with the i-STAT are systematically lower compared to the concentrations determined by the Centaur.

## Introduction

Cardiac troponin I (cTnI) is a sensitive and specific biomarker for the detection of myocardial injury in humans. In recent years cTnI has been evaluated in ruminant species with cardiac diseases such as pericarditis (1), endocarditis (2, 3), as well as in neonates with congenital cardiac defects (4, 5), non-cardiac disorders (6, 7), nutritional muscular dystrophy (8, 9), myocarditis (10–12), umbilical abscessation (13), envenomation (14), pregnancy toxemia (7, 15, 16), ruminal acidosis (17, 18), and endotoxemia (19). Similarly, infection with *Theileria annulata* in cattle is associated with increased circulating cTnI (20). Multiple toxicities have been reported to increase cTnI in ruminant species, ranging from plants such as Rayless Goldenrod (*Isocoma pluriflora*), (21) to anti-inflammatories such as diclofenac (22), as well as the ionophore antibiotics, such as monensin (23–25). As such, any increased blood concentration of cTnI is an indicator of myocardial injury and has been associated with an adverse clinical outcome in human patients (26), as well as downer cattle (27). There are currently multiple clinical applications for cTnI in bovine practice.

Ionophore antibiotics such as monensin are widely used in cattle industries for control of coccidiosis and improved feed efficiency (28). Monensin is considered safe in cattle when it is fed at recommended dosages, although it may become unsafe if higher concentrations are fed or mixing errors occur (24, 29). Because of the mechanism of interfering with the cation membrane transport, the ionophores can cause cell death by destabilizing cell membranes, particularly in skeletal and cardiac muscle (30). Traditional methods of diagnosis of ionophore concentrations, such as liquid chromatography/mass spectrometry (31), while sensitive, are ill-suited for a rapid or on-farm diagnosis. As such, testing for elevated cTnI concentrations could hasten time to diagnosis for monensin toxicity in cattle.

Multiple cTnI assays are currently available, and method analytical variation can lead to clinically relevant discordance of measured cTnI concentrations (32). This variation is due to the lack of standardized calibration material, the use of different detection antibodies, and differences in reagent formulations and assay parameters (32). The obtained cTnI values from one assay to another can differ by a factor of 10 or even more (33); therefore, the measured cTnI concentrations are often not comparable between assay manufacturer. Poor inter-assay agreement was found when three different assays were compared for the detection of canine cTnI (34). In the same study an up to 19-fold difference among analyzers was detected (34). Complete standardization can only be achieved if all assay manufacturers would utilize the same antibodies, which is a difficult goal because of their intellectual property and economic impact (35). A general recommendation is that the assay antibodies should only recognize the stable part of the cTnI protein that does not form complexes with troponin T and troponin C (36).

The ADVIA Centaur TnI-Ultra immunoassay (Centaur) represents a validated method for detection of circulating cTnI concentration between 0.2 and 30 ng/mL in cattle (37). While useful, this analyzer is not portable, and as such does not provide the utility to ambulatory bovine practitioners that a point-of-care analyzer would. The i-STAT®1 (i-STAT) immunoassay represents a point-of-care assay that has use in ambulatory bovine practice and has been assessed for evaluating circulating cTnI concentrations in normal cattle (38). A reference interval for healthy cattle of 0.0–0.036 ng/mL (median 0.02 ng/mL) has also been determined for the i-STAT (39). While high precision was identified with the i-STAT compared to the Centaur for healthy cattle (38), the agreement between the two tests for cattle with myocardial injury is currently unknown.

The objective of this study was to compare the analytical performance of the previously validated (37) ADVIA Centaur TnI-Ultra immunoassay with the point-of-care (POC) i-STAT®1 immunoassay in the detection of cTnI in blood of cattle. Serum samples from healthy cattle and cattle with myocardial injury due to experimentally induced monensin toxicosis were used for this study. We hypothesized that both assays would be able to detect bovine cTnI but that there would be a clinically relevant difference in measured cTnI concentration between the two assays for healthy cattle as well as cattle with induced myocardial injury.

## Materials and Methods

### Animals

This study was approved by the Institutional Animal Care and Use Committee (IACUC) at the Ohio State University, Columbus, OH. The healthy control group consisted of 30 apparently healthy dairy cattle (26 Holstein, 4 Jersey). The animals were considered healthy based on history and physical examination findings including cardiac and thoracic auscultation. Twenty Holstein cows were pregnant (between 40 and 215 days) and their average daily milk yield was 24.5 ± 6 kg. The remaining cows were in the dry off period. The mean estimated body weight was 544 ± 71 kg based on body condition score and height of the animal assessed by two independent investigators and averaged as previously described (40). Twenty-five cows were <5 years old and five cows were >5 years old. A 10 ml blood sample was collected from either the jugular or the coccygeal vein directly into a serum vacutainer. Blood was left to clot at 23°C for a maximum of 45 min before the tubes were centrifuged at 2,800 rpm (1,500 g) for 20 min. Serum was removed and divided into two aliquots and frozen at −20°C within 4 h of collection. Samples were analyzed within 2 days of collection (37).

The study group consisted of 10 apparently healthy non-pregnant, non-lactating dairy cows (six Jersey, four Holstein) which were used in a previous study conducted by the authors (24). Mean body weight was 494 kg (SD 85 kg) and seven were <5 years old. All animals were healthy based on physical examination findings, blood work, electrocardiogram, and echocardiography. Administration of a single oral dose of monensin was performed via suspension in 300 ml of water and flushed with additional 500 ml of water to ensure all residual monensin within the tube was administered to the cow. The tube was further flushed between administrations. An indwelling jugular catheter was placed in all cows prior to administration of the monensin. Two cows received 30 and 40 mg/kg monensin, while the remaining eight cows received a dose of 50 mg/kg monensin. A blood sample was collected from all cows at 4, 6, 8, 12, 20, 24, 36, 48, 72, and 80 h (30 mg/kg and 40 mg/kg monensin) and at 12, 24, 36, 48, 72, 96, 120, and 144 h (50 mg/kg monensin). However, due to early removal from the study due to death or euthanasia, a total of only 92 blood samples were taken. Sample collection and handling was done in the same way as described for the healthy control group.

### Sample Preparation and Analysis

Frozen samples were sent out in batches to a commercial laboratory or evaluated in-house for measurement of the cTnI concentration with the ADVIA Centaur TnI-Ultra immunoassay and the i-STAT immunoassay, respectively. Only a single measurement of the analyte concentration per sample was performed. The ADVIA Centaur immunoassay is a three-site sandwich assay using direct chemiluminometric technology for the detection of free and complexed cTnI. It includes one polyclonal goat and two monoclonal mouse antitroponin-I-antibodies. These capture antibodies recognize the amino acid sequences 87–91 and 41–49 located in the stable region of the human cTnI protein (41). A previous validation study of the assay performed in our laboratory revealed sufficient analytical performance for the detection of bovine cTnI (37). The i-STAT®1 is a 10-min point-of-care assay, which uses a two-site ELISA method. Monoclonal anti-cTnI antibodies (caprine, murine) recognize the amino acid sequences 41–49 and 88–91 of the cTnI protein (42). The lower limit of detection of the Centaur assay based on manufacturer's validation for humans is at 0.02 ng/ml with a reportable range of 0.0 to 50 ng/ml. However, previous study identified cTnI concentrations of 0.01 ng/mL in bovine samples, with excellent linearity of samples of 0.5–30 ng/mL (37). The lower limit of detection of the i-STAT assay based on manufacturer's validation for humans is at 0.02 ng/ml with a reportable range of up to 50 ng/ml (43).

### Statistical Analysis

Thirty serum samples of the control group and 92 serum samples of the study group were analyzed for association and agreement between the methods. Association was evaluated via linear regression analysis. Agreement between methods including bias and limits of agreement was determined by use of the Bland-Altman method (44). Based on previous observations (24) that monensin-induced myocardial necrosis as detected and quantified by histopathology was present when serum cTnI concentration was 1.04 ng/ml or above, two groups were formed. The first group comprised serum samples with cTnI <1 ng/ml (determined by Centaur assay), but above the limit of detection (0.02 ng/mL) and the second group serum samples with cTnI concentrations ≥1 ng/ml (determined by Centaur assay), and methods were compared separately for both groups. Samples were analyzed for correlation overall, and then as separate groups. If the Pearson r value was <0.975, a Deming regression analysis was performed as previously described (45). Performance goals were evaluated with the 95% confidence intervals of the slope and intercept including 1 and 0 (respectively) being considered acceptable, as previously described (46). Commercially available statistical software (Prism 8.3.0, GraphPad Software LLC) was used.

## Results

**Figure 1:**
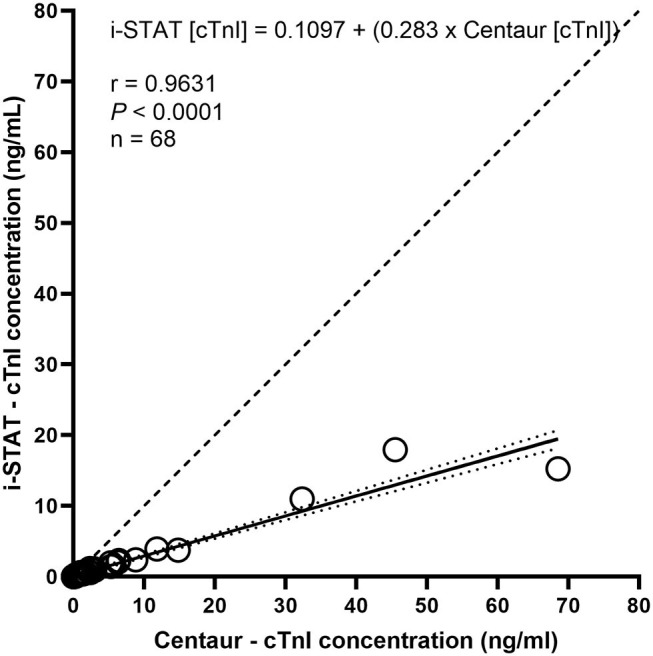
Linear regression of cTnI concentrations as determined by i-STAT and Centaur analyzers. Circles indicate concentrations as determined by i-STAT (Y axis) and Centaur (X axis). The solid line represents regression line with dotted lines recommending 95% confidence intervals for the regression line. The dashed line represents a line of identity.

The total number of samples where serum cTnI was measured by both methods was 111. In one cow only the Centaur cTnI analysis was available. This measurement was removed from the analysis. For 43 measurements both samples were below the limit of detection of the assays. Sixty-eight samples had a cTnI concentration above the lower limit of detection of the Centaur assay and 55 samples had measurable concentrations via the i-STAT. Sixty-eight (98.5%) of i-STAT cTnI values were lower than the Centaur cTnI values. One (1.5%) measurement was equal with both assays. None (0%) of samples had i-STAT measurements higher than Centaur values. The results of linear regression analysis of all 111 dual method samples are shown in Figure 1. The Pearson r was 0.9631 (95% confidence interval: 0.94 to 0.98), with the regression equation being represented as: i-STAT [cTnI] = 0.1097 + (0.283 x Centaur [cTnI]). The 95% confidence interval of the slope being: 0.262 to 0.301; and the 95% confidence intervals of the intercept being: −0.100 to 0.331.

### Serum cTnI Concentrations <1 ng/mL

**Figure 2:**
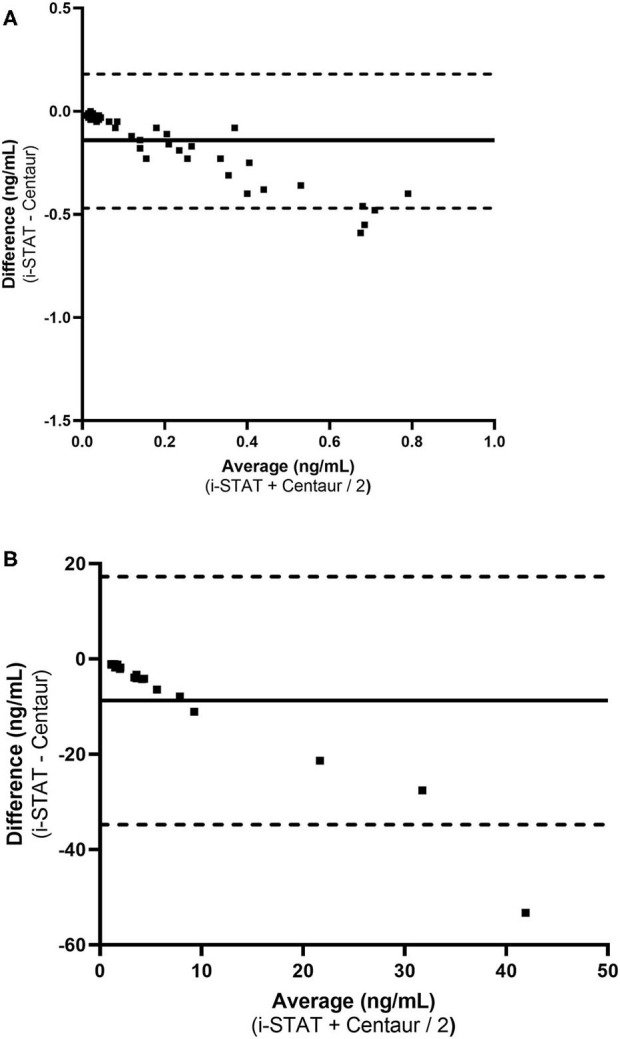
Bland-Altman plots demonstrating measurement difference as a function of measurement for low and high serum cTnI concentrations. **(A)** cTnI < 1.0 ng/mL (*n* = 47). **(B)** Average cTnI ≥ 1.0 ng/mL (*n* = 18). The horizontal solid line in the center of each graph represent the mean difference (bias) between the methods and the dotted lines represent the limits of agreement between the two methods of measurement.

Ninety-three samples had a serum cTnI concentration below 1 ng/ml (range: 0.01–0.99 ng/mL) as determined by the Centaur assay. Of these 47 had values above the limit of detection (0.02 ng/mL) for the Centaur assay. These 47 samples had a Pearson r of 0.9656 (95% confidence interval: 0.94 to 0.98). Deming regression of these 47 samples revealed an equation represented as: i-STAT [cTnI] = −0.008273 + (0.4862 × Centaur [cTnI]). The 95% confidence intervals of the slope being: 0.421 to 0.552; and the 95% confidence interval of the intercept being: −0.0195 to 0.003. Figure 2A demonstrates the Bland Altman plot for the 47 samples. Bias for these samples was −0.14 ng/mL (± 0.16), indicating a negative proportional bias, with the 95% limits of agreement of −0.47 to 0.18 to ng/mL.

### Serum cTnI Concentrations ≥1 ng/mL

Eighteen samples had a serum cTnI concentration ≥1 ng/ml (range: 1.65–68.4 ng/mL) as determined by the Centaur assay. For these 18 samples the range for the i-STAT was 0.53–17.94 ng/mL. These 18 samples had a Pearson r of 0.9499 (95% confidence interval: 0.87 to 0.98). Deming regression of these 18 samples revealed an equation represented as: i-STAT [cTnI] = 0.37 + (0.2762 × Centaur [cTnI]). The 95% confidence intervals of the slope being: 0.0538 to 0.499; and the 95% confidence intervals of the intercept being: −1.095 to 1.833. Figure 2B demonstrates the Bland Altman plot for each analyzer for all 18 samples. Bias for these samples was −9.81 ng/mL (± 13.26), indicating a negative proportional bias, with the 95% limits of agreement of −34.80 to 17.19 ng/mL.

The 95% confidence intervals of the slope and intercepts of the cTnI concentrations of <1.0 ng/mL and >1.0 ng/mL would not be considered acceptable. While the confidence intervals of the slopes did not include 1, both intercepts did include 0. However, as reported by Flatland et al. both of those criteria must be met for an “acceptable” method comparison (46).

## Discussion

This study compared serum cTnI concentrations from both healthy cattle as well as cattle with experimentally induced cTnI elevation as determined by two different analyzers—a hand-held point-of-care device and a fully automated laboratory analyzer. The results of this study indicate that the concentrations of cTnI determined by each analyzer were not equal, as the i-STAT point-of-care analyzer consistently yielded concentrations less than the previously validated Centaur analyzer. This discrepancy has to potential to lead to underestimation of serum cTnI concentrations in cattle. Better agreement between methods was noted when samples with <1 ng/mL cTnI concentration were analyzed compared to samples with concentrations ≥1 ng/mL indicating a proportional bias. This difference may not be clinically important considering the suggested reference interval of serum cTnI in cattle of 0.00–0.05 ng/mL as determined by the point-of-care analyzer (39) and cattle with myocardial disease often being diagnosed with much higher serum cTnI concentrations (24). However, such discrepancies may lead to false negative as well as false positive results when applied to a given patient where accurate diagnosis of myocardial disease is clinically relevant (19, 24, 37). Because of this clinicians should consider method specific reference intervals for evaluating cTnI in cattle.

The agreement between the two devices compared in this study has been established for bovine samples with low cTnI concentrations (38). Similar to the findings for samples of low concentration (<1.0 ng/mL cTnI) in our study, an investigation of the i-STAT point-of-care analyzer using bovine plasma samples with spiked cTnI concentrations ≤ 1.0 ng/mL it was found that blood cTnI concentrations determined by the i-STAT were not different than the cTnI concentrations evaluated with the Advia Centaur immunoassay (38). Test precision was relatively high with a coefficient of variation <20% (38). However, when assays were compared in our study the bias was notably different for samples with cTnI ≥ 1.0 ng/mL (bias = −8.749 ng/mL) when compared to the bias of samples with cTnI <1.0 ng/mL (bias = −0.013 ng/mL). As such, the i-STAT consistently underestimated true serum cTnI. As such, a negative proportional bias for the iSTAT vs. the Centaur was observed. Considering that the Centaur has been validated and thus can be considered accurate, it is concluded that serum cTnI determined with the iSTAT will be falsely low, potentially leading to incorrect diagnoses of myocardial damage in cattle if i-STAT results are interpreted with cutoffs and reference intervals specific to the Centaur.

The increased difference between i-STAT and Centaur results with increasing cTnI concentration is an example of proportional bias. As opposed to fixed bias, where the differences between two methods remains constant, the differences in proportional bias increase with the value of the concentration being measured (47). Proportional bias was recently identified a human study where three point-of care cTnI assays were compared to one central laboratory assay when evaluating high-sensitivity cTnI (48). Proportional bias can occur from matrix effect and calibration functions in analytical assays (49). It is possible that this difference could be due to the lack of standardized calibration material and harmonization amongst cTnI assays that has been previously described (50–52).

Even with the systematic underestimation by the point-of-care analyzer, the i-STAT may still have clinical utility for bovine practice, in particular when cTnI is normal or only marginally increased. However, potential inaccuracy should be considered in animals with considerable cTnI elevations and if this assay is used in animal research. In humans and dogs there is a close correlation between the magnitude of elevated blood cTnI concentrations and the severity of myocardial cell damage (53, 54). Similar findings have been reported in cattle (24). Increased cTnI concentrations have also been identified in other ruminant species with myocardial damage including goats (11, 23) and sheep (3, 12). In a study of histopathologically confirmed and morphometrically graded monensin toxicity in cattle, cTnI concentrations ranged from 0.4 to 39.0 ng/mL (median: 16.0 ng/mL) (24). Similarly, a 15 day old calf that died of severe myocarditis had a circulating antemortem cTnI concentration of 37.24 ng/mL (55). Downer dairy cows with a cTnI concentration of >0.7 ng/mL have worse prognosis within 7 days of testing (27). Therefore, while analytical discrepancies may be largely ignored in some populations of cows, in particular when cTnI concentrations are low or below the lower limit of detection of the assay, they may become more important in animals with elevated cTnI where estimation of the severity of myocardial damage is clinically relevant.

Several limitations of this study need to be considered. The number of samples available for analysis was adequate but not extensive. Analysis of cTnI were not done in duplicate and results averaged; this may have led to additional error. A high sensitivity cTnI assays was not used for comparison, and the lower limit of detection of both assays was relatively high which may have influenced the conclusions of this study. Also, an animal model of myocardial cell damage induced by monensin was used. Potential interference of monensin with the cTnI assays and generation of undetectable cTnI complexes with monensin cannot be excluded.

## Conclusions

In clinically normal cattle, concentrations of circulating cTnI as determined by the i-STAT point-of-care assay were similar to the concentrations reported by a previously validated method (Centaur) for determination of serum cTnI in cattle. Results from serum samples from cattle with experimentally-induced monensin cardiotoxicity and elevated cTnI showed a significant negative proportional bias for TnI when measured with the iStat compared to the Centaur. Further research is warranted to evaluate the analytical performance of the i-STAT in cattle with cardiac disease. Despite its limitations, the i-STAT point-of-care analyzer may be useful for ambulatory practitioners in the detection of myocardial cell damage in cattle with cardiopulmonary disease.

## Data Availability Statement

The raw data supporting the conclusions of this article will be made available by the authors, without undue reservation.

## Ethics Statement

The animal study was reviewed and approved by Institutional Animal Care and Use Committee, The Ohio State University. Written informed consent was obtained from the owners for the participation of their animals in this study.

## Author Contributions

AV and KS contributed to study design, sample collection, sample analysis, statistical analysis, and manuscript construction. JS contributed to sample collection, statistical analysis, and manuscript construction. All authors approved this manuscript.

## Conflict of Interest

AV was employed by the company Gold Coast Veterinary Services. The remaining authors declare that the research was conducted in the absence of any commercial or financial relationships that could be construed as a potential conflict of interest.
